# Access barriers to maternal healthcare services in selected hard-to-reach areas of Zambia: a mixed methods design

**DOI:** 10.11604/pamj.2021.40.4.28423

**Published:** 2021-09-02

**Authors:** Chris Mweemba, Miriam Mapulanga, Choolwe Jacobs, Patricia Katowa-Mukwato, Margaret Maimbolwa

**Affiliations:** 1 Department of Health Policy, Systems and Management, School of Public Health, University of Zambia, Lusaka, Zambia,; 2 Department of Public Health, University of Lusaka, Lusaka, Zambia,; 3 Department of Epidemiology and Biostatistics, School of Public Health, University of Zambia, Lusaka, Zambia,; 4 School of Nursing Sciences, University of Zambia, Lusaka, Zambia

**Keywords:** Access, maternal health, antenatal care, availability, affordability, acceptability

## Abstract

**Introduction:**

poor access to maternal health services is a one of the major contributing factors to maternal deaths in low-resource settings, and understanding access barriers to maternal services is an important step for targeting interventions aimed at promoting institutional delivery and improving maternal health. This study explored access barriers to maternal and antenatal services in Kaputa and Ngabwe; two of Zambia´s rural and hard-to-reach districts.

**Methods:**

a concurrent mixed methods approach was therefore, undertaken to exploring three access dimensions, namely availability, affordability and acceptability, in the two districts. Structured interviews were conducted among 190 eligible women in both districts, while key informant interviews, in-depth interviews and focus group discussions were conducted for the qualitative component.

**Results:**

the study found that respondents were happy with facilities´ opening and closing times in both districts. By comparison, however, women in Ngabwe spent significantly more time traveling to facilities than those in Kaputa, with bad roads and transport challenges cited as factors affecting service use. The requirement to have a traditional birth attendant (TBA) accompany a woman when going to deliver from the facility, and paying these TBAs, was a notable access barrier. Generally, services seemed to be more acceptable in Kaputa than in Ngabwe, though both districts complained about long queues, being delivered by male health workers and having delivery rooms next to male wards.

**Conclusion:**

based on the indicators of access used in this study, maternal health services seemed to be more accessible in Kaputa compared to Ngabwe.

## Introduction

Maternal mortality remains a major health problem in low-resource settings due to weak health systems and poor health service delivery [1-3]. For instance, in 2017, 94% of the 295,000 global maternal deaths occurred in low-income countries, with two thirds of these deaths occurring in sub-Saharan Africa (SSA) [4]. One of the major contributing factor to maternal deaths, in these settings, is the low access to healthcare services, especially in rural areas [5,6]. Maternal services, such as antenatal care (ANC), provide an opportunity for prevention and early detection of maternal conditions such as anaemia, malaria, hypertension and other medical conditions affecting maternal health, and thereby improving pregnancy outcomes [7]. Delayed and poor access to services is associated with increased maternal, fetal and infant mortality and morbidity [8]. Access is, therefore, an important aspect of service delivery and has been receiving increased attention due to its importance in health policy [9]. The debate, however, is what constitutes “access” and how it can be operationalized. To make the concept of access operational, this study adopted the McIntyre Access Framework which defines access as the “degree of fit” between clients and the health care system [9]. The framework defines three distinct dimensions of access, i.e. availability, affordability and the acceptability.

The availability dimension examines whether appropriate services are provided in the right place and at the right time to meet the needs of the population [9]. Availability is further broken down into four elements; the location of services in relation to the location of clients, the “ability and willingness” of providers to offer services desired by the people, the ‘degree of fit´ between the facility operating hours and the time clients need services, and finally the relationship between the type of services offered and the health needs of the users [9]. The affordability dimension is association with the costs of health care services and the users´ ability to pay these costs [9-11]. The acceptability dimension, on the other hand, is the fit between providers and clients expectations, characteristics, practices, etc. towards each other [9-11]. The acceptability dimension is closely linked to trust, because once providers´ expectations, characteristics, practices, beliefs and align with those of users, trust is enhanced and access is promoted [12].

Despite the improvement in access to maternal health services in Africa over the years, it has remained relatively low in rural parts of Zambia compared to other countries in the region [13]. In Zambia, only 65% of rural women had four or more ANC visits in 2018 [14], an indication that more effort is required for the country to achieve universal access to maternal healthcare services. This study, therefore, aimed at exploring the availability, affordability and acceptability of maternal services in Zambia´s two rural and hard-to-reach districts of Kaputa and Ngabwe. An understanding of access barriers is an important step towards designing interventions to improve maternal health and reduce maternal mortality [4].

## Methods

**Study setting:** the study was conducted in Zambia´s two rural and hard-to-reach districts of Kaputa and Ngabwe, located in the county´s worst performing provinces of Northern and Central provinces, respectively, in as far as maternal indicators are concerned. Data collection was done between June and July, 2016.

**Research design:** a convergent parallel design was conducted to address the study objectives. Both quantitative and qualitative data were collected at the same time and results interpreted together [15]. A mixed methods approach offers potential for identifying, exploring and understanding accessibility of maternal health services in rural Zambia.

**Quantitative component:** a multi-stage sampling technique was employed for the quantitative study. First, purposive sampling was used to select the two worst performing provinces in maternal health indicators, based on the 2013/14 Zambia Demographic and Health Survey (ZDHS) [16], followed by the selection of the worst performing district in the selected provinces. Cluster sampling was then employed to select three communities per district, after which households with either pregnant women or women who had given birth in the five years preceding this study, were identified from a household listing exercise. A random sample of 95 households were selected per district using simple random sampling. Randomization was utilized to address sample selection bias [17]. The sample size was determined using the prevalence formula [18]. Quantitative data was collected using a pretested structured questionnaire, and information collected included participants´ demographic information and questions measuring indicators of availability, affordability and acceptability of maternal health services. Specifically, availability was measured by travel time, mode of transport used to get to a health facility, and the convenience of facility opening and closing times. Affordability was measured by the expenditure on maternal services, medication, transport, accommodation, food, communication and paying someone to take care of the children at home. Employment status was used as an indicator of respondents´ ability to pay. Acceptability was measured by how respondents felt about length of queues, health workers´ attitudes and respect, and cleanliness of facilities.

**Qualitative component:** qualitative data was collected through four (4) Focus Group Discussions (FGDs) per district with women utilizing maternal health services. In addition, a total of eight (8) Key Informant Interviews (KIIs) per district were conducted; four (4) with Traditional Birth Attendants (TBAs) and four (4) with the facility in-charges. The FGDs were conducted in the community while KIIs were done from the facilities. Participants for the FGDs were identified from the household listing exercise while TBAs were identified with the help of facility-in-charges. The triangulation methods were used to enhance the validity of the findings.

**Data analysis:** the quantitative data was analysed using STATA version 11 for windows [19]. Descriptive statistics were computed to describe the access indicators relating to availability, affordability and acceptability of maternal health services. Qualitative interviews were transcribed verbatim, while those conducted in local language were transcribed then translated to English. The transcripts were checked for completeness and read thoroughly to ensure that no information was lost due to translation. The transcripts were then analysed thematically following already predetermined themes generated from the three access dimensions. The data analysis was conducted with the aid of Nvivo [20].

**Ethical consideration:** ethical approval was obtained from the University of Zambia Biomedical Research Ethics Committee (UNZABREC) (Reference Number: 937-2020) and permission from the Zambia National Health Research Authority (ZNHRA). Additional permission was obtained from respective provincial and district health offices. Informed consent was obtained from all participants prior to the interviews.

## Results

**Quantitative findings:** a total of 219 respondents were interviewed in the two districts with slightly more interviews in Kaputa (n=113). Respondents in Kaputa were on average older, more likely to be pregnant and to make at least four maternal related visits during the current or previous pregnancy, compared to those in Ngabwe. Respondents in Kaputa also had better school attendance and employment status. Table 1 summarizes these findings.

**Table 1:** participants’ demographic characteristics

	Kaputa (N=113)		Ngabwe (N=106)	
**Age**				
Mean age	28.8		27.8	
Minimum age	18		18	
Maximum age	50		46	
	**Frequency**	**Percent**	**Frequency**	**Percent**
**Current pregnant**				
No	83	73.5%	87	82.1%
Yes	30	26.6%	19	17.9%
**Number of ANC visits in current/previous pregnancy**				
One	6	5.3%	10	9.5%
Two	13	11.5%	22	20.8%
Three	28	24.8%	24	22.6%
Four	65	57.5%	45	42.5%
Don't know	1	0.9%	5	4.7%
**Highest level of education**				
No schooling	16	14.2%	17	16%
Primary	93	82.3%	74	69.8%
Secondary	3	2.7%	15	14.2%
University/college	1	0.8%	-	-
**Currently employed**				
No	95	84.1%	102	96.2%
Yes	18	15.9%	4	3.8%
**Head of household currently employed**				
No	85	75.2%	88	83%
Yes	20	17.7%	7	6.6%
Missing	8	7.1%	11	10.4%
**Borrowed money**				
No	85	75.2%	92	87.6%
Yes	28	24.8%	13	12.4

**Availability of maternal services:** the results revealed that, significantly more respondents in Kaputa, compared to Ngabwe, reported visiting facilities closest to their homes (78.6% versus 63.46%; P=0.0142). Majority of respondents in both districts were happy with facility opening and closing times. With regards to travel time, respondents in Ngabwe spent significantly more time traveling to facilities than those in Kaputa (mean time of 136 minutes versus 105 minutes; P=0.0028; 6 hours versus 5 hours maximum travel time). The difference in travel time between the districts existed despite most respondent in Ngabwe (78.3%) using bicycles while majority in Kaputa (70.27%) walking to facilities. Similarly, pregnant women in Kaputa received significantly more ANC home visits than their counterpart in Ngabwe. Table 2 presents quantitative results on availability of services.

**Table 2:** availability of maternal health services

	Kaputa (%)	Ngabwe (%)	P-Value
**Availability indicators**			
Facility visited is closest to home	78.6%	63.5%	0.0142
Facility opening hours convinient	90.9%	94.3%	0.3451
Facility closing hours convinient	92.7%	92.5%	0.9375
**Mode of transport to the facility**			
Bicycle	27.0%	78.3%	<0.001
By foot	70.3%	12.3%	<0.001
Minibus	2.7%	5.7%	0.2747
Taxi/cab	-	1.9%	-
Other	-	1.9%	-
**Home visits during pregnancy**			
Yes	15.2%	6.7%	0.0457

**Qualitative findings:** the qualitative findings revealed that maternal facilities were sparsely located in both districts making it challenging for women to travel safely and deliver from the facilities. A health worker from Kaputa had the following to say on distances to facilities: *“these mothers have to walk all that distance just to come and access maternal health services and the furthest being 12 km, if am not mistaken, so you would find that if someone does not have transport they prefer to stay at home and not to come for antenatal services”* (KHW 01). Similarly, a health worker from Ngabwe had the following remarks regarding distance to the facilities: *“you would find that a mother is 20 kilometres away from the centre, even if she is supposed to deliver from the health facility, you would find that by the time they are organizing transport or even as they are coming to the facility they would have even already delivered on the way, so that hinders them”* (NHW 03).

Apart from distance, respondents also complained about the bad state of the roads, especially during the rainy season, making the use of other modes of transport, such as bicycles, motorbikes and cars, very challenging. A woman in Ngabwe said the following: *“even if you have a bicycle or motorbike….. the problem is the bad state of the road and how to move on it because you find a lot of mud on the way which can make the bicycle get stuck so the only solution is to deliver from home”* (NW 08). A TBA in Ngabwe had similar sentiments: *“even vehicles get stuck but even if you are in the vehicle and in labour you may even deliver due to the bumps, so it´s hard”* (NTBA 04). Other respondents cited lack of transport as another barrier to facility visits. A male respondent from Kaputa had the following concern: *“transportation is a problem….vehicles don´t come here”* (km 03). The quantitative and qualitative findings suggest an intimate relationship between institutional delivery, location of facilities, state of the roads and transportation.

### Affordability of maternal health services

**Quantitative findings:** the quantitative results on affordability revealed that respondents in Kaputa spent more at the last facility visit, with a maximum amount of K500 (approximately $49*) having been spent at the last visit compared to K400 (approximately $39) in Ngabwe (Median amounts of K0 and K15 (approximately $1.5), respectively). However, an examination of actual cost drivers revealed mixed findings. For instance, while respondents in Kaputa spent more on hospital fees, those in Ngabwe spend more on buying medicines. Similarly, respondents in Ngabwe spent more on accommodation and buying food during facility visits, while those in Kaputa spent more on phone related costs and paying someone to take care of children when visiting maternal health services. Based on this study´s definition of ability to pay, there was significantly higher employment rates in Kaputa than in Ngabwe. Note, however, that respondents in Kaputa still borrowed significantly more than those in Ngabwe to finance their maternal related activities (24.78% versus 12.38%; p=0.0193). Table 3 summarizes these quantitative findings on affordability of services.

**Table 3:** affordability of maternal health services

	Kaputa (%)			Ngabwe (%)		
**Expenditure on maternal health services**	**Median amount (Kw)**	**Mean amount (Kw)**	**Maximum amount (Kw)**	**Median amount (Kw)**	**Mean amount (Kw)**	**Maximum amount (Kw)**
Money spent on last ANC visit	0	49.7	500	15	28.0	400
Amount spent on hospital fees	0	8.2	150	0	2.3	35
Paying for medicines	0	0.4	40	0	1.6	150
Transport	0	4.3	100	0	6.9	50
Accommodation	0	0.6	50	0	2.6	200
Food	0	3.9	40	10	12.9	200
Phone	0	0.6	35	0	0.5	10
Pay someone to take care of children	0	1.5	100	0	0.3	10

Note that the mounts are in Zambian kwacha

**Qualitative findings:** notwithstanding the fact that maternal health services are offered free of charge in Zambia, respondents on this study reported paying “unofficial” fees to TBAs during delivery, with respondents in Kaputa reporting this practice more. A respondent from Kaputa reported the following: *“… if you come here and you are asked to pay Kw25 (approximately $2.5) by the TBAs then what´s the purpose of the clinic and why do they have to assign the TBAs there and the health workers are not there?”* (KW03). However, in-depth discussions with women revealed that the TBA fee was paid whether or not a woman was escorted by a TBA: *“even if you come with your mother, you will still be instructed to go and look for the TBA, until you produce the Kw25 (approximately $2.5), ..that really hurts me. It means there is no clinic you just as good as the one delivering from the village”* (KW#10). Note that respondent from Ngabwe also acknowledged paying TBAs, and that this payment was a token of appreciation for the service rendered. One respondent had the following remarks: *“everyone who delivers from the clinic had to pay the TBAs something…Kw50 (approximately $4.9) for others and Kw100 (approximately $9.8) for first timers”* (NW04).

### Acceptability of maternal healthcare services

**Quantitative findings:** when it came to acceptability of maternal health services, the majority of respondents in both districts reported long queues, with respondents in Ngabwe significantly more likely to report long queues than those in Kaputa (83% versus 67.6%, p=0.0085). In addition, 28% and 40% (p=0.0685) of respondents in Kaputa and Ngabwe, respectively, complained that healthcare workers were too busy to listen to their problems. Similarly, 38% and 61% (p<0.001) of respondents in Kaputa and Ngabwe, respectively, complained of healthcare workers not treating them with sufficient respect. Though not statistically significant, 31% of respondents in Kaputa and 23% in Ngabwe reported dirty waiting area and toilets in the facilities they visited. Generally, majority of respondents in both districts were satisfied with maternal health services, with significantly more respondents in Kaputa being satisfied with services than those in Ngabwe (96% vs 63%; p<0.001). Table 4 summarizes these quantitative findings.

**Table 4:** acceptability of maternal health services

	Kaputa (%)	Ngabwe (%)	P-Value
**Acceptability indicators**			
The queues at the ANC clinic are too long	67.6%	83.0%	0.0085
Health workers are too busy to listen to clients problems	27.9%	39.6%	0.0685
Some staff do not treat clients with sufficient respect	38.2%	61.3%	<0.001
The ANC facility (including waiting area and toilets) is dirty	30.6%	22.9%	0.1978
Generally satisfied with ANC services	96.4%	63.2%	<0.001

**Qualitative findings:** the qualitative interviews revealed insightful information relating to acceptability of maternal services in the two districts. Respondents were asked about factors that may affect their use of services, and some women in Kaputa were not comfortable being attended to by young male health providers during labour and delivery. One woman expressed herself as follows: *“…being attended to is sometimes difficult, especially if the person is male and young, we are uncomfortable especially adults that´s why many end up delivering in the village”* (KW 05). A health worker in Kaputa confirmed that having male attendants was a potential reason for some women opting to deliver from home: *“…you find that there are only males so there again with the setup of the area, and the lower education levels of the people, they would rather be discouraged to be seen by the male and stay at home,…so that one is a problem, for deliveries you find that they won´t be coming concurrently”* (KHW 03).

In addition, some women in Kaputa were concerned about the lack of privacy in the ANC facilities. One woman remarked: *“the delivery room here is next to the male ward and so we have problems with that because what goes on there….male patients can hear”* (KW 06). A male respondent in Ngabwe felt that some facilities lacked “safer spaces for child deliveries”, and said the following: *“…it´s not just a room, let it be spacious like 3 rooms, where you have a waiting area, delivery room and where to keep babies when they are born. In English it´s pre-natal and where a woman should be put after delivery as she recovers and where new born babies are kept. Can that be done?”* (NM 02). Generally, however, services seemed to be more appealing to the respondents in Kaputa than those in Ngabwe.

## Discussion

The findings are discussed in line with the three access dimensions but are synthesized in the conclusion.

**Availability of services:** respondents reported covering long distances to facilities, with some taking over 100 minutes to get to maternal health services, despite majority of them using facilities closest to their homes, and also using bicycles and other transport modalities to get to facilities. Distance may be one of the reasons why only 65% of rural women had made at least four ANC visits in Zambia [14]. The Institute for Health Metrics and Evaluation (IHME) [21] also acknowledges the low levels of ANC coverage in Zambia. Other studies have also found distance to be an important barrier preventing women from utilizing maternal services [22,23]. Choulagai and others´ (2013), for instance, found that distance prevented women from seeking skilled birth services in Nepal. Similar findings were reported in Ghana [22] and Malawi [24]. Related to distance, our study and other studies found poor road network, high transport costs and the general lack of transport as barriers to using maternal health services [25-27].

It is worth noting, however, that over 90% of the respondents in our study were happy with the facilities´ operating hours. This is encouraging considering that other countries in the region have reported incompatibility between facility operating times and clients´ preferred time [28]. Service availability is an important aspect of access, and is one of the six key outcome areas for the health systems strengthening framework, aimed at achieving Universal Health Coverage (UHC), and the Sustainable Development Goals (SDGs) [29].

**Affordability of services:** while it is expected that service users would spend money on transport, food, communication and sometimes on accommodation, it was unusual that they were required to pay TBAs. This highlights the importance and influence of TBAs within the maternal health care system of low-resource settings [30,31]. However, if financial protection and institutional deliveries are critical policy goals, then pregnant women should be protected from any form of financial cost when seeking institutional delivery [32]. The financial costs highlighted in this study may seem insignificant, but can be very substantial considering the economic vulnerability of research participants [33]. Service related costs can discourage institutional deliveries, as shown in Bangladesh, Pakistan and South Africa [27,34], and can also cause households to resort to strategies such as borrowing and selling of assets [35]; practices that have additional undesirable effects on households´ future wellbeing [36,37]. There should be deliberate efforts, therefore, to facilitate institutional delivery by ensuring that women don´t have to pay for the service, or at least to encourage non-cash payments such as crop yields, clothing and other in-kind payments [34,38]. The employment rates are very low in most limited-resource settings to sustain financial costs [39], and expecting poor pregnant women to pay for institutional deliveries can create inequities in service use [40].

**Acceptability of services:** long queues were a common occurrence in the facilities of both districts, with the problem reported more in Ngabwe. This highlights the imbalance between service provision and the number of women seeking maternal related services, which inevitably results in overcrowding and long waiting times in facilities [41,42].

Overcrowding has the potential to limits the privacy and safe spaces for delivering mothers [23,43]. Overcrowding in facilities also results in healthcare worker burnout [44,45] which compromises quality of care [46]. This may result in mistrust, between clients and service providers, and can negatively affect patient satisfaction [47,48]. The eroding of trust between clients and providers compromises the very essence of access as a fit between system and community factors [9,12]. In addition, congestion in facilities can result in impaired communication and bitter relationships between staff and clients [45,49], with the potential to make clients feel disrespected.

## Conclusion

Distances to facilities, bad roads and poor transport system are important availability barriers to institutional delivery. Some notable affordability barriers to facility delivery, included; requiring pregnant women to be accompanied by TBAs to the facilities, and pay for TBA services. In terms of acceptability, respondents highlighted lack of privacy in the facilities and having males assisting with deliveries, as key barriers.

**Limitations:** this study was done in two of Zambia´s rural districts, and generalizing these findings to other districts should be done with caution due to the heterogeneity of districts in the way maternal healthcare services are distributed.

**Funding:** this work was supported by the Norwegian Programme for Capacity Development in Higher Education and Research for Development (NORHED).

### What is known about this topic

- Distance and cost of services are important determinants of institutional deliveries.

### What this study adds

- TBAs are still an important cadre in maternal healthcare system of rural communities of Zambia;
- This study has also revealed the existence of “TBA fees” which have the potential to create socioeconomic related inequalities in service access.
